# Prognostic Impact and Prevalence of Cachexia in Patients With Heart Failure: A Systematic Review and Meta‐Analysis

**DOI:** 10.1002/jcsm.13596

**Published:** 2024-10-30

**Authors:** Konstantinos Prokopidis, Krzysztof Irlik, Mirela Hendel, Julia Piaśnik, Gregory Y. H. Lip, Katarzyna Nabrdalik

**Affiliations:** ^1^ Department of Musculoskeletal Ageing and Science, Institute of Life Course and Medical Sciences University of Liverpool Liverpool UK; ^2^ Liverpool Centre for Cardiovascular Science at University of Liverpool Liverpool John Moores University and Liverpool Heart & Chest Hospital Liverpool UK; ^3^ Students' Scientific Association by the Department of Internal Medicine, Diabetology and Nephrology in Zabrze, Faculty of Medical Sciences in Zabrze Medical University of Silesia Katowice Poland; ^4^ Doctoral School, Department of Internal Medicine, Diabetology and Nephrology, Faculty of Medical Sciences in Zabrze Medical University of Silesia Katowice Poland; ^5^ Department of Clinical Medicine, Danish Center for Health Services Research Aalborg University Aalborg Denmark; ^6^ Department of Internal Medicine, Diabetology and Nephrology, Faculty of Medical Sciences in Zabrze Medical University of Silesia Katowice Poland

**Keywords:** cachexia, heart failure, mortality, muscle wasting, weight loss

## Abstract

**Background:**

Cachexia, defined as the combination of weight loss, weakness, fatigue, anorexia and abnormal biochemical markers based on Evans' criteria, is known to exacerbate the prognosis of heart failure (HF) patients. This systematic review and meta‐analysis investigates the prognostic impact and prevalence of cachexia, as defined by Evans' criteria, in patients with HF.

**Methods:**

PubMed, Cochrane Library, Scopus and Web of Science were searched from inception until December 2023, including HF patients for whom the Evans' criteria were applied to explore the prevalence and prognostic impact of cachexia. This study employed a meta‐analyses using the random‐effects model and inverse‐variance method that was adhered to the revised 2020 PRISMA guidelines for systematic reviews and meta‐analyses (CRD42023446443).

**Results:**

Six prospective or retrospective studies of 2252 patients with HF were included, whereby all‐cause mortality was significantly greater in patients with cachexia with low heterogeneity among studies (HR: 1.60, 95% CI 1.31–1.95, *p* < 0.001; *I* ^2^ = 0%). For the studies that used full, uniformly defined Evans' criteria, among 1844 patients, mortality remained greater in patients with cachexia (HR: 1.58, 95% CI 1.27–1.97, *p* < 0.001; *I* ^2^ = 0%). In a subgroup analysis among 1714 of HF with reduced ejection fraction, the results were consistent (HR: 1.57, 95% CI 1.28–1.92, *p* < 0.001; *I* ^2^ = 0%). Additionally, 10 studies comprising 2862 patients indicated a 31% risk of cachexia in HF (95% CI 21–43%, *I* ^2^ = 94%).

**Conclusions:**

Cachexia is an independent predictor for increased all‐cause mortality among patients with HF with a notable prevalence of 31%. Interventions aiding in improving fatigue, anorexia and exercise capacity could help improve the quality of life of this clinical population.

## 1 Introduction

Cachexia, a complex metabolic syndrome characterized by involuntary weight loss, muscle wasting and altered metabolic and biochemical pathways, has emerged as a significant determinant in the prognosis of heart failure (HF). The relationship between cachexia and HF introduces a multifaceted dynamic that extends beyond the traditional understanding of HF as solely a cardiovascular disorder.

Cachexia imposes a profound impact on the overall health status of individuals with HF, contributing to a decline in skeletal muscle mass, compromised functional capacity and metabolic derangements [1]. Of note, the aetiology of cachexia in HF is multifactorial, involving inflammatory processes, neurohormonal imbalances and altered protein metabolism, leading to a state of increased catabolism that significantly influences patient outcomes [1]. In particular, ghrelin resistance has been suggested as a possible mediator, that may explain, in part, appetite losses in patients with cachexia [2, 3], while a decrease in neuropeptide Y (NPY) and proopiomelanocortin (POMC) expression has been implicated in Transforming growth factor beta (TGF‐b) mechanisms [4], affecting central appetite regulation. Interestingly, some evidence has also proposed a potential impact of tumour necrosis factor‐alpha (TNF‐a), exacerbating bitterness in taste bud cells that could also be a contributor to reduced food intake [5]. In addition, cachexia in HF is often accompanied by a persistent inflammatory state characterized by elevated levels of TNF‐a and interleukin‐6 (IL‐6) [6]. Chronic states of inflammation contribute to systemic endothelial dysfunction and impaired immune responses, factors that could increase cardiovascular complications, thromboembolism and subsequent mortality [7, 8]. Impaired metabolic homeostasis via incremental skeletal muscle mass losses may hamper glucose and insulin sensitivity (and vice versa) [9], leading to reduced exercise capacity, loss of physical strength and function, and overall independence [7]. The difficulties occurring through secondary sarcopenia, a major contributor to reduced quality of life, may also be exacerbated by anorexia that encompasses reduced energy intake linked to malnutrition [8]. The connection among systemic inflammation, malnutrition and muscle wasting could potentially elevate mortality risk as well as loss of independence and functional capacity.

**FIGURE 1:**
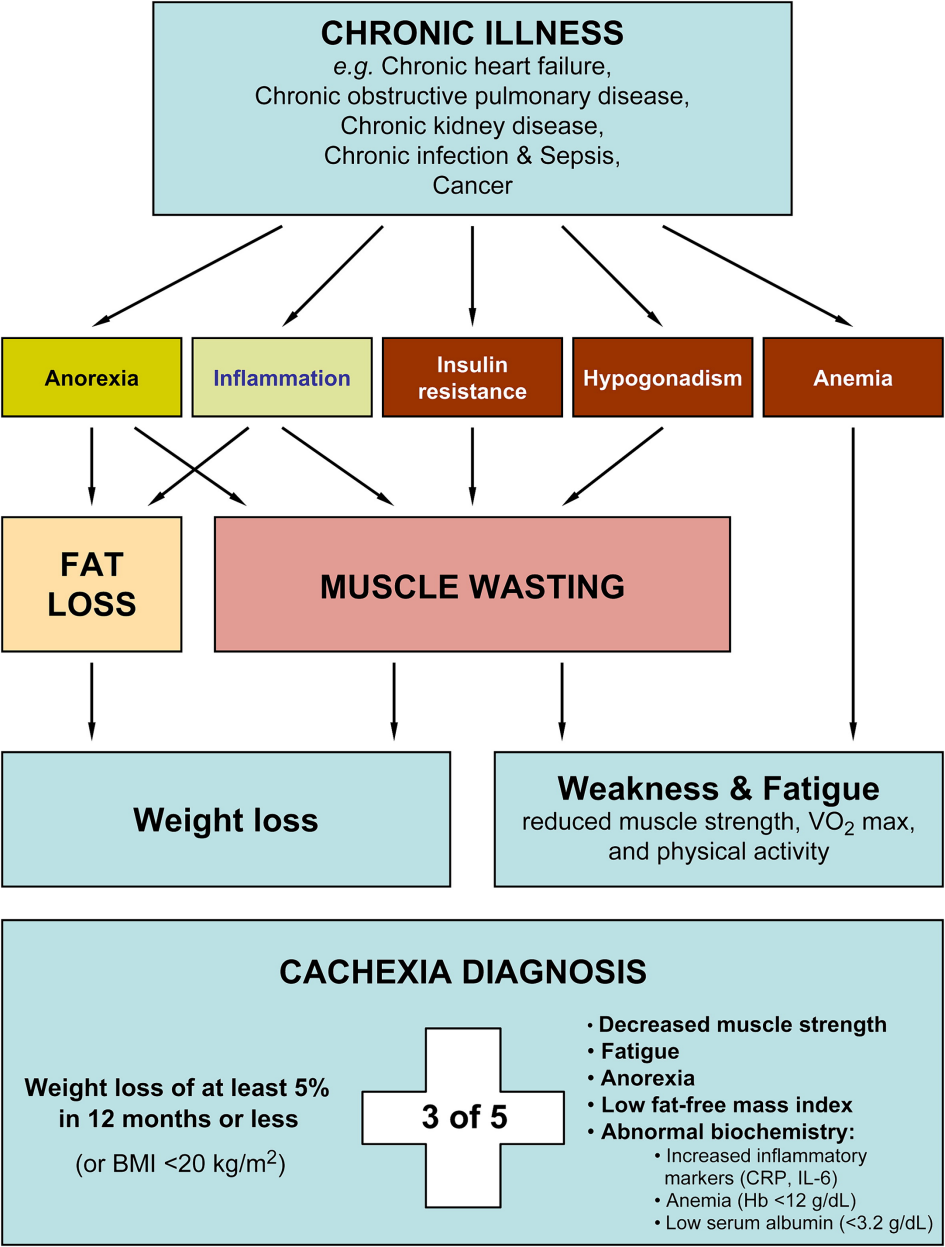
Definition of cachexia based on Evans' criteria [15].

Higher mortality rates associated with cachexia [10] may be attributed to increasing weight loss and muscle wasting [11] that perpetuate reductions in quality of life and survival rates [12]. Previous meta‐analyses have found differing results on mortality in HF primarily derived from studies examining involuntary weight loss (usually minimum 5% weight loss in the last 6 to 12 months). More specifically, hazard ratios (HR) of 1.74 [95% confidence interval (CI) 1.35–2.24] [13] and 3.84 (95% CI 2.28–6.45) [14] with increased heterogeneity among studies were recently reported. Although rapid weight loss in this clinical population may be a significant contributor to losses of lean mass and functionality, cachexia is a multifactorial phenomenon that underpins deficits in multiple domains in addition to weight losses, as mentioned above. Considering that weakness, fatigue, anorexia and abnormal biochemical markers are key features in patients with cachexia, Evans et al. attempted to provide a consensus definition to capture a more prominent clinical feature of cachexia (Figure 1) [15].

As researchers delve deeper into elucidating the mechanisms governing cachexia in HF, the identification of potential therapeutic targets becomes imperative. Interventions targeting systemic inflammation, muscle wasting and metabolic imbalances hold promise in mitigating the adverse impact of cachexia on HF prognosis. An established definition approaching clinical models may support the integration of personalized and multidisciplinary approaches and may pave the way for innovative strategies aimed at improving patient outcomes and quality of life.

The aim of this systematic review and meta‐analysis is to unravel the prognostic impact of cachexia based solely on Evans' criteria in patients with HF and determine its prevalence, which could raise clinical awareness, advocating for its treatment by healthcare professionals.

## 2 Methods

### 2.1 Search Strategy

Four independent reviewers (KP, MH, JP and KI) searched PubMed, Scopus, Web of Science and Cochrane Library from inception until December 2023, using the search strategy outlined in the Table S1. Additionally, references and citations of the articles assessed for eligibility were searched.

### 2.2 Eligibility Criteria

Studies were included based on the following criteria: (i) cohort studies, (ii) individuals >18 years old with HF irrespective of type and clinical setting (i.e., outpatients and inpatients) and (iii) cachexia assessment was performed based on Evans' criteria. Published articles were excluded if they (i) were reviews, letters, in vivo or in vitro experiments or commentaries and (ii) were not published as a full text and in English. PICO (Patient population, Exposure, Comparison, Outcome) criteria can be found in Table S2.

Definition of cachexia according to Evans' criteria:

Weight loss of at least 5% in 12 months or less, and three of the following criteria:

- decreased muscle strength (lowest tertile)
- fatigue
- anorexia
- low fat‐free mass index
- abnormal biochemistry:

- increased inflammatory markers: C‐reactive protein (CRP) (>5.0 mg/L), interleukin‐6 (IL‐6) (>4.0 pg/mL)
- anaemia (haemoglobin <12 g/dL)
- low serum albumin (<3.2 g/dL)

In cases where weight loss history cannot be determined a BMI <20.0 kg/m^2^ is sufficient.

### 2.3 Data Extraction

Two investigators (KI and MH) extracted data independently, including the name of first author, year of publication, country of origin, study design, definition of cachexia, frequency of cachexia, patient characteristics such as age, sample size, sex, body mass index (BMI), left ventricular ejection fraction rate (LVEF%) and reported co‐morbidities. Disagreements between authors were resolved by a third investigator (KP).

### 2.4 Risk of Bias Assessment

The quality assessment of the included studies was performed via the Quality Assessment Tool for Observational Cohort and Cross‐Sectional Studies provided by the National Institutes of Health (NIH). This tool is comprised of 14 questions and was developed not to assign a numerical score, but to conduct a critical appraisal of the internal validity of the studies, hence an overall quality rating of ‘Good’, ‘Fair’ or ‘Poor’. If a study was labelled as ‘Poor’, it was considered as having high risk of bias.

### 2.5 Certainty of the Evidence

The evaluation of evidence certainty was performed utilizing the Grading of Recommendations, Assessment, Development and Evaluations (GRADE) framework. We modified our approach according to Iorio et al. [16] for a prognosis meta‐analysis, assessing the impact of cachexia on all‐cause mortality. In the field of prognosis, a body of longitudinal cohort studies initially provides high confidence. The certainty of evidence was adjusted for factors such as risk of bias, inconsistency, indirectness, imprecision and publication bias, the latter of which could not be assessed due to the limited number of studies included.

### 2.6 Statistical Analysis

Outcomes between patients with HF and with or without cachexia were compared using HR. Quantitative data were treated as continuous and prevalence of cachexia among patients with HF was calculated as a proportion of cases with cachexia and total study population. For studies presenting interquartile ranges (IQR), the formula ‘standard deviation (SD) = width of IQR/1.35’ approximated missing SDs [17]. Statistical significance was determined through the random‐effects model and inverse‐variance method.

Evaluation of heterogeneity among outcome measures across studies employed a 95% confidence interval (95% CI) overlap, Cochran's *Q* (chi‐square test), and *I* ^2^. Data were categorized as having low (30%–49%), moderate (50%–74%) or high (75% and above) heterogeneity. Subgroup analysis based on HF with reduced ejection fraction rate was performed. Sensitivity analyses were performed to account for higher number of reported co‐morbidities among patients with HF and cachexia versus HF without cachexia and studies that may had partially followed Evans' criteria as part of cachexia assessment. The synthesis of meta‐analyses was conducted through Review Manager (RevMan 5.4.1) software and R software using ‘meta’ package (version 4.3.1, R Foundation for Statistical Computing, 2020, Vienna, Austria), with a significance threshold set at *p* < 0.05.

Finally, in case studies did not report data based on multivariate analyses, they were included in a narrative synthesis.

## 3 Results

The literature search employed resulted in 4951 publications. Following the exclusion of duplicates and abstracts, 61 articles were identified as potentially eligible for inclusion. Of these 61 studies, 41 were excluded because they did not adhere to Evans' criteria to define cachexia to evaluate its prognostic impact, one study was excluded due to identical cohort with more recent included study in this systematic review and meta‐analysis [18], five studies were not published as full‐text articles, two were not original reports, while another study used only BMI (<20 kg/m^2^) and one biochemical marker below its suggested cut‐off according to the Evans' criteria [15].

**FIGURE 2:**
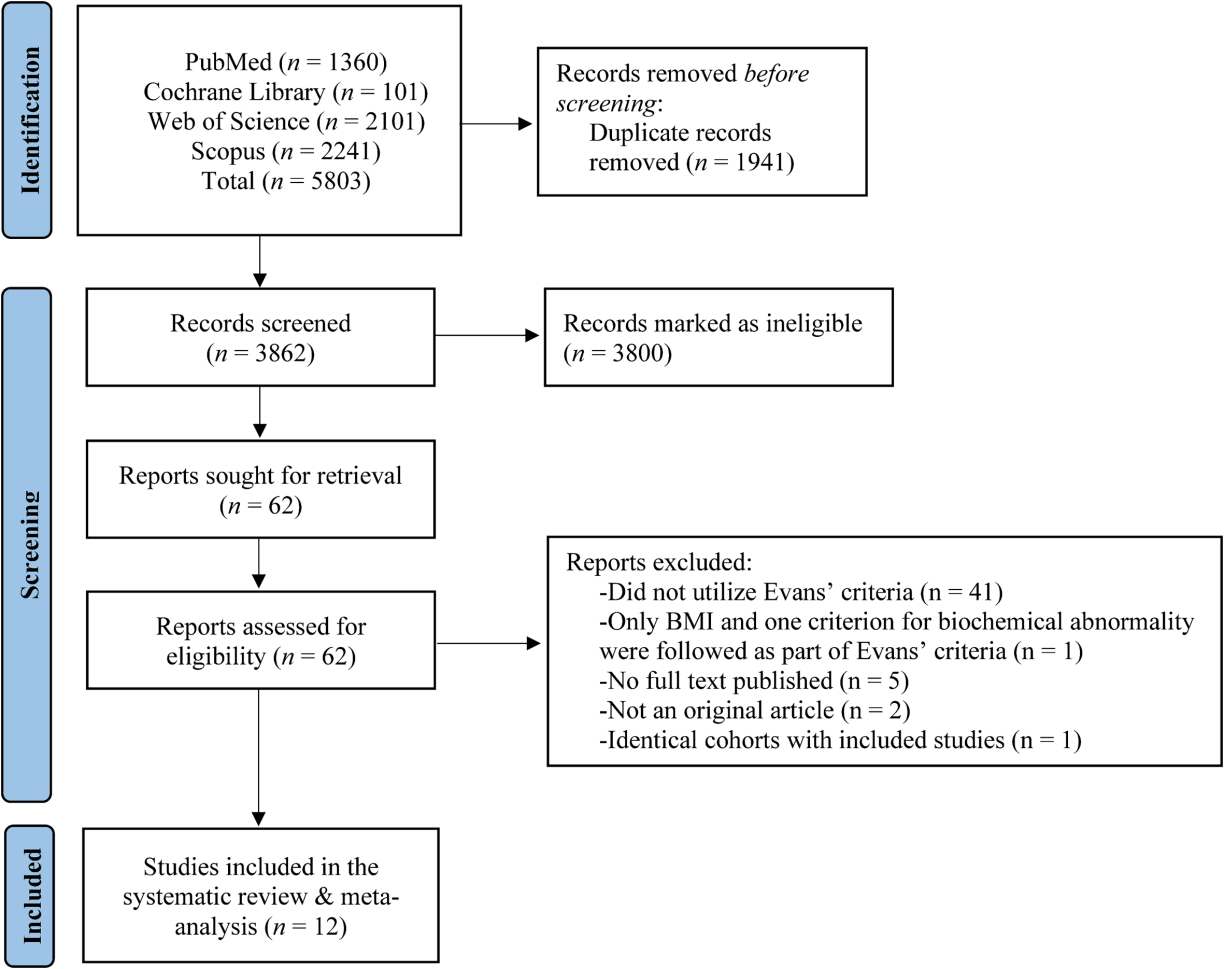
Flowchart of the literature search.

In total, six studies [10, 19, 20, 21, 22, 23] evaluated the impact of cachexia on all‐cause mortality via Evans' criteria, including three with prospective and three with retrospective design, while for the prevalence of cachexia 10 studies were utilized, using Evans' criteria [10, 21, 22, 23, 24, 25, 26, 27, 28, 29] (Figure 2). Detailed characteristics of the included studies are shown in Table 1.

**TABLE 1:** Study and participant characteristics of the included studies defining cachexia according to Evans criteria in patients with heart failure.

Study, year	Country	Total *n* (M/F)	Cachectic				Noncachectic			
			*n* (M/F)	Age	BMI	LVEF %	*n* (M/F)	Age	BMI	LVEF %
Armas et al., 2023	United States	37	14 (12/2)	57.5 (47–64)	23.1 (21.2–28.0)	—	23	—	—	—
Maekawa et al., 2023	Japan	1306 (745/561)	463 (255/208)	82 (76–86)	19.2 (17.4–21.7)	45.0 (31.5–61.0)	843 (490/353)	80 (74–86)	21.8 (19.9–24.2)	45.0 (32.2–60.0)
Carson et al., 2022	United Kingdom	200 (131/69)	30 (19/11)	75.6 ± 11.7	21.8 ± 4.4	—	170 (112/58)	74.2 ± 13.1	29.9 ± 7.4	—
Sobieszek et al., 2021a	Poland	91 (91/0)	40 (40/0)	75 ± 12.5	28.27 ± 6.6	34 ± 13.5	51 (51/0)	72 ± 14	29.61 ± 5.0	41 ± 14.5
Sobieszek et al., 2021b	Poland	157 (92/65)	74 (40/34)	78.5 (70.0–86.0)	26.9 (24.2–30.1)	40.0 (25.0–52.7)	83 (52/31)	68.5 (59.0–78.0)	30.0 (26.4–33.5)	45.0 (30.0–55.0)
Sobieszek et al., 2020	Poland	66 (0/66)	34 (0/34)	80 (12)	28.02 ± 6.22	42 ± 13.0	32 (0/32)	77 (9)	31.64 ± 6.41	48 ± 9.0
Morishita et al., 2021	Japan	370 (240/130)	88 (52/36)	76.0 (65.7, 82.0)	19.1 (17.4, 20.6)	51.0 (39.0, 61.0)	282 (188/94)	69.0 (60.0, 75.0)	23.1 (21.4, 25.1)	52.0 (40.0, 65.0)
Valentova et al., 2016	Germany	165 (135/30)	29 (25/4)	70.3 (61.1–76.4)	23.7 (22.3–27.1)	30.0 (20.0–33.1)	136 (110/26)	68.4 (60.9–74.5)	27.8 (25.5–32.3)	35.0 (30.0–40.0)
Letilovic et al., 2013	Croatia	42	30	All: 63.4	22.84 ± 4.46	—	12	All: 63.4	24.40 ± 3.23	—
Szabo et al., 2014	Germany	111 (100/11)	18 (18/0)	71 (8)	23 (4)	28 (5)	93 (82/11)	63 (11)	27 (4)	37 (10)
Saitoh et al., 2017	Germany	—	—	—	—	—	—	—	—	—
Melenovsky et al., 2013	Czech Republic	408 (343/65)	78 (70/8)	60 (11)	25.6 (4.3)	24 (5.3)	330 (274/56)	59 (11)	28.3 (4.6)	26 (6.7)

*Note:* Data are expressed as mean (standard deviation) or median (IQR).

### 3.1 Cachexia and All‐Cause Mortality: Multivariate Analyses

**FIGURE 3:**
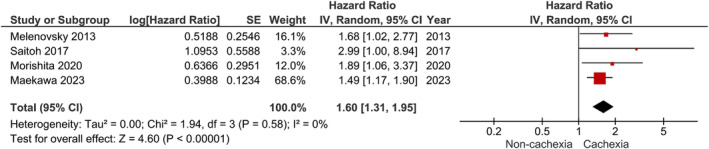
Effects of cachexia on all‐cause mortality in patients with heart failure.

All‐cause mortality was significantly greater in patients with cachexia based on low heterogeneity among studies (HR: 1.60, 95% CI 1.31–1.95, *p* < 0.01; *I* ^2^ = 0%) (Figure 3). Following a subgroup analysis based on HF with solely reduced ejection fraction, results were similar (HR: 1.57, 95% CI 1.28–1.92, *p* < 0.01; *I* ^2^ = 0%) (Figure S1).

Similar findings were observed after a sensitivity analysis in which only studies meeting the full Evans' criteria were included (HR: 1.58, 95% CI 1.27–1.97, *p* < 0.01; *I* ^2^ = 0%) (Figure S2) and the exclusion of one study based on increased risk of bias (HR: 1.56, 95% CI 1.26–1.94, *p* < 0.01; *I* ^2^ = 0%) (Figure S3). Confounders adjusted for in mortality analyses along with effect estimates of all studies are presented in Table S3.

### 3.2 Cachexia and All‐Cause Mortality: Univariate Analyses

Considering that no adjustments for covariates were made in two studies [19, 22], we decided to not conduct a meta‐analysis. Therefore, a narrative synthesis reporting their results was the recommended option by all investigators. In one study, although 95% CI values were not reported, cachexia in patients with HF had a prognostic impact of 6.89 [19], while in another study 4.14 (95% CI 1.99–8.63) (*p* < 0.01) [22].

### 3.3 Prevalence of Cachexia in Patients With Heart Failure

**FIGURE 4:**
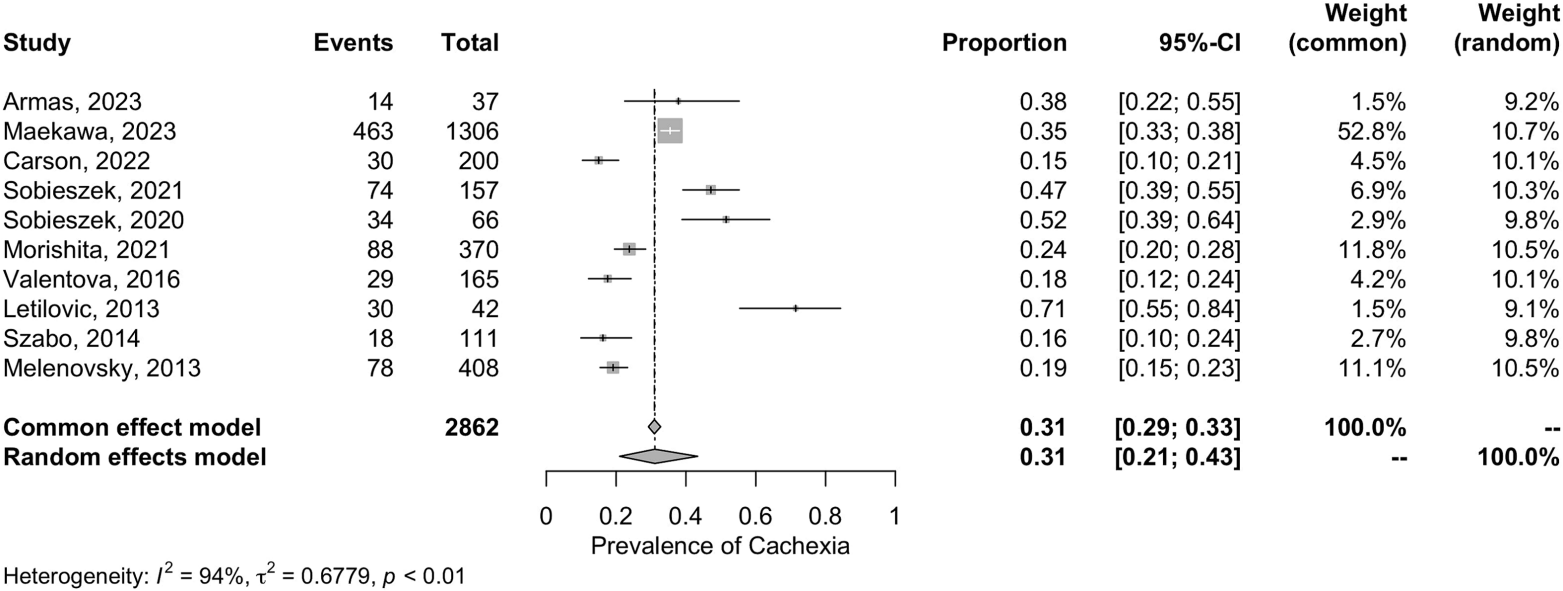
Prevalence of cachexia defined by Evans' criteria among patients with HF.

Patients with HF (*n* = 2862) had 31% risk of having cachexia using Evans' definition (k = 10; 95% CI 21–43, *p* < 0.01; *I* ^2^ = 94%) (Figure 4). Based on sensitivity analyses excluding studies with increased risk of bias, results remained similar, cachexia was observed in 38% of the participants (Evans' definition; *n* = 2144; k = 6; 95% CI 23–56, *p* < 0.01; *I* ^2^ = 95%; Figure S4).

### 3.4 Meta‐Regression Analyses on the Prevalence of Cachexia in Heart Failure

Heterogeneity observed among studies was further investigated through meta‐regression analyses using age and BMI as covariates. Regarding the risk of cachexia and noncachexia in patients with HF, age and BMI did not act as confounders (age: *r* = 0.0253, SE = 0.058, 95% CI −0.09 to 0.14, *z* = 0.43, *p* = 0.66; BMI: *r* = 0.2290, SE = 0.122, 95% CI −0.01 to 0.47, *z* = 1.88, *p* = 0.06) (Table S4).

### 3.5 Risk of Bias Assessment and Certainty of the Evidence

Overall, among studies that assessed mortality as an outcome, only one study seemed to have a good overall quality [10]. Two studies were considered ‘Fair’ [20, 23], while three studies were evaluated as ‘Poor’ [19, 21, 22]. Among studies with data on cachexia prevalence, three were judged as ‘Good’ [10, 27, 28], three as ‘Fair’ [23, 25, 29], and four were deemed as ‘Poor’ quality studies [21, 22, 24, 26]. A detailed table depicting the results of risk of bias assessment is shown in Tables S5 and S6. Certainty of the evidence was considered moderate for studies exploring the impact of cachexia on all‐cause mortality (Table S7), while low for studies assessing the prevalence of cachexia in HF (Table S8).

## 4 Discussion

In this systematic review and meta‐analysis, our results show a significantly greater risk of all‐cause mortality in patients with HF and cachexia using Evans' criteria, with a low heterogeneity. These findings were identical following subgroup analysis based on HFrEF. Furthermore, our results demonstrated a prevalence of cachexia of 31% using Evans' definition. These findings displayed high heterogeneity, which may be explained by BMI differences among studies.

### 4.1 Prevalence of Cachexia and Its Prognostic Impact

The aforementioned results depict a skewed relationship between the risk of cachexia following HF. Considering that Evans' criteria to define cachexia consists of at least three criteria related to decreased muscle strength, fatigue, anorexia, low fat‐free mass and/or altered serum levels CRP, haemoglobin and albumin in conjunction with weight loss, it is expected that less patients would meet these criteria. Albeit slightly lower risk of cachexia under Evans' criteria, it is worth noting that a prevalence of 31% may be alarming particularly in patients with lower BMI that would correlate with lower baseline appendicular lean mass, fat‐free mass and a greater risk of malnutrition [30].

In our analysis, we applied the stringent Evans' criteria for cachexia to reduce heterogeneity. Despite this, high heterogeneity persisted, suggesting influences beyond diagnostic criteria. Variations in heart failure populations, such as differences in baseline weight, HF severity, co‐morbidities and treatment regimens, as well as inconsistent methods for evaluating Evans criteria components (e.g., muscle strength and fat‐free mass) likely contributed to this continued variability. This highlights the complex, multifaceted nature of cachexia in HF.

Decreased muscle strength and fat‐free mass are major prognostic factors of all‐cause mortality in cachexia and HF rather than weight loss alone [10]. The study by Maekawa et al. demonstrated a high prevalence of cachexia among patients with HF, reaching approximately 36%. Nevertheless, this study included hospitalized patients with stable HF and a median age above 80 that could amplify the presence of cachexia [10]. In addition, given the overlap between cachexia and sarcopenia, the coexistence of these two conditions may exacerbate mortality risk [18]. Under conditions of involuntary weight loss, it is critical to consider the crosstalk between HF and muscle wasting [1] in a way that patients with HF may be more prone to greater losses of skeletal muscle due to secondary sarcopenia. Therefore, our results reinforce the need for implementing effective rehabilitation strategies in this patient group especially in more vulnerable states (i.e., hospitalized).

In our meta‐analysis, when inspecting the impact of cachexia on mortality, we observed low heterogeneity (*I* ^2^ = 0%), even though the studies varied widely in demographics and clinical conditions. This uniformity might stem from the consistent use of the multifactorial cachexia definition proposed by Evans et al. [15], a framework that is becoming increasingly common in clinical research. This methodological consistency across studies likely contributes to the apparent strong and stable impact of cachexia on mortality, suggesting that the effects of cachexia may indeed be generalizable across different clinical environments. Nevertheless, we must consider the limitations of the *I* ^2^ index with caution, especially given the small number of studies involved in this analysis. The *I* ^2^ value, in such cases, might not adequately represent the true variability among the studies, potentially understating the real heterogeneity [31].

### 4.2 Strengths and Limitations

This study utilized data based on a more complete profile of cachexia, including uniform Evans' criteria that capture a more clinically representative model of this condition, however, the variability in reporting standards for components of cachexia definition such as low muscle strength, fatigue, and low fat‐free mass index, often without detailed methodological descriptions, may have influenced our findings. In addition, our analysis was characterized by low heterogeneity among studies (*I* ^2^ = 0%) pertinent of cachexia's prognostic impact on all‐cause mortality. However, the studies included employed multivariate analysis for which the confounders used were not identical among studies, which is a limitation. In addition, we included four longitudinal cohorts, considering the strict criteria as an inclusion criterion that limited the number of eligible studies. Finally, only three studies were considered of having a low risk of bias out of six included in this study. In terms of the studies included related to the prevalence of cachexia in HF, our study was prone to limitations pertaining to the cross‐sectional nature of the data utilized for analysis, the increased heterogeneity among studies, the increased risk of bias from multiple studies incorporated in our analyses, and the lack of studies allowing for distinction between reduced and preserved ejection fraction rates regarding the risk of cachexia.

## 5 Conclusions

Cachexia is a significant prognostic factor for all‐cause mortality in this population with a mean cachexia risk of 31% using Evans' criteria. Assessment of multiple markers related to rate of weight loss, physical function, anorexia, and biochemical parameters are critical for clinicians in identifying patients who are at risk of cachexia. Interventions in clinical and nonclinical settings, aiming to improve symptoms of fatigue, anorexia and exercise capacity could help improve patients' quality of life under this condition.

## Ethics Statement

The authors of this manuscript certify that they comply with the ethical guidelines for authorship and publishing in the *Journal of Cachexia, Sarcopenia and Muscle* [32].

## Conflicts of Interest

The authors declare no conflicts of interest.

## Supporting information


**Figure S1** Effects of cachexia on all‐cause mortality in patients with heart failure and reduced ejection fraction.

**Figure S2** Effects of cachexia based on full Evan's criteria on all‐cause mortality in patients with heart failure.

**Figure S3** Effects of cachexia on all‐cause mortality in patients with heart failure excluding studies with increased risk of bias.

**Figure S4** Prevalence of cachexia defined by Evans’ criteria among patients with HF after exclusion of studies with high risk of bias

**Table S1** Key terms employed in the screening of the literature search.

**Table S2** PICO characteristics of research questions in this systematic review.

**Table S3** Study outcomes and variables adjusted for in the multivariable analyses.

**Table S4** Meta‐regression analyses of patients with heart failure and cachexia vs. patients with heart failure without cachexia using Evans’ criteria.

**Table S5** Risk of bias assessment for studies evaluating impact of cachexia on mortality.

**Table S6** Risk of bias assessment for studies utilized to assess prevalence of cachexia using Evans criteria.

**Table S7** Summary of findings table for the impact of cachexia on all‐cause mortality in patients with HF.

**Table S8** Summary of findings table for the risk of cachexia among patients with HF.

**Data S1** Supporting Information.

## Data Availability

Data are available upon request.
