# Supplementary figures and images for: Prognostic Impact and Prevalence of Cachexia in Patients With Heart Failure: A Systematic Review and Meta‐Analysis
Source: J Cachexia Sarcopenia Muscle. 2024 Oct 30;15(6):2536–43. doi: 10.1002/jcsm.13596 (PMC11634528; doi:10.1002/jcsm.13596)

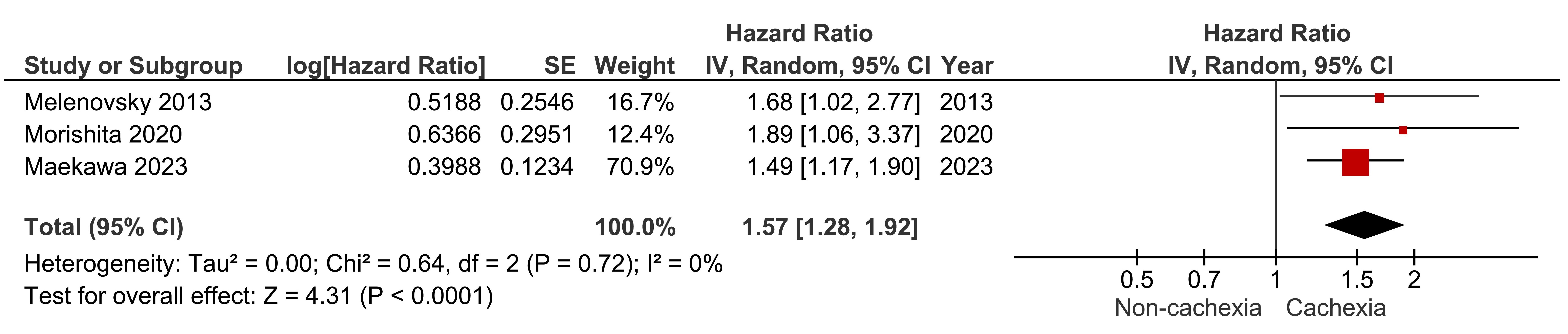

Supplement: Supplementary file 1 — Figure S1 Effects of cachexia on all‐cause mortality in patients with heart failure and reduced ejection fraction. [file JCSM-15-2536-s008.jpg]

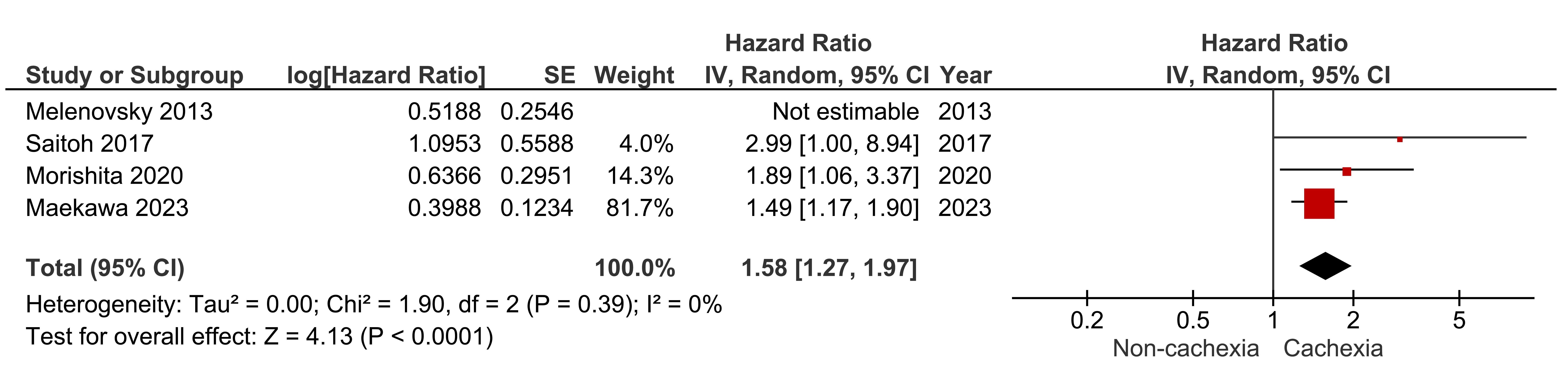

Supplement: Supplementary file 2 — Figure S2 Effects of cachexia based on full Evan's criteria on all‐cause mortality in patients with heart failure. [file JCSM-15-2536-s006.jpg]

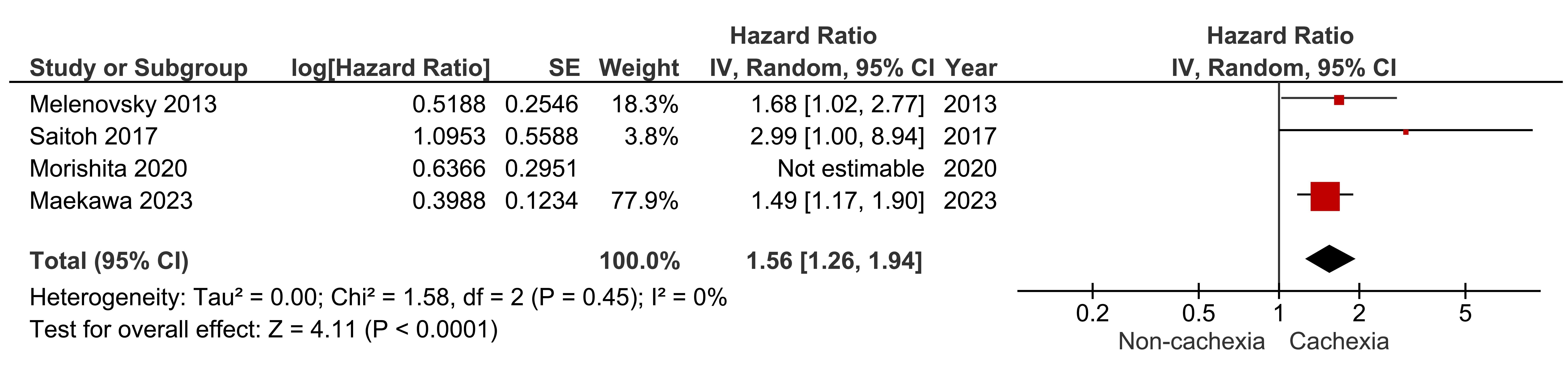

Supplement: Supplementary file 3 — Figure S3 Effects of cachexia on all‐cause mortality in patients with heart failure excluding studies with increased risk of bias. [file JCSM-15-2536-s012.jpg]

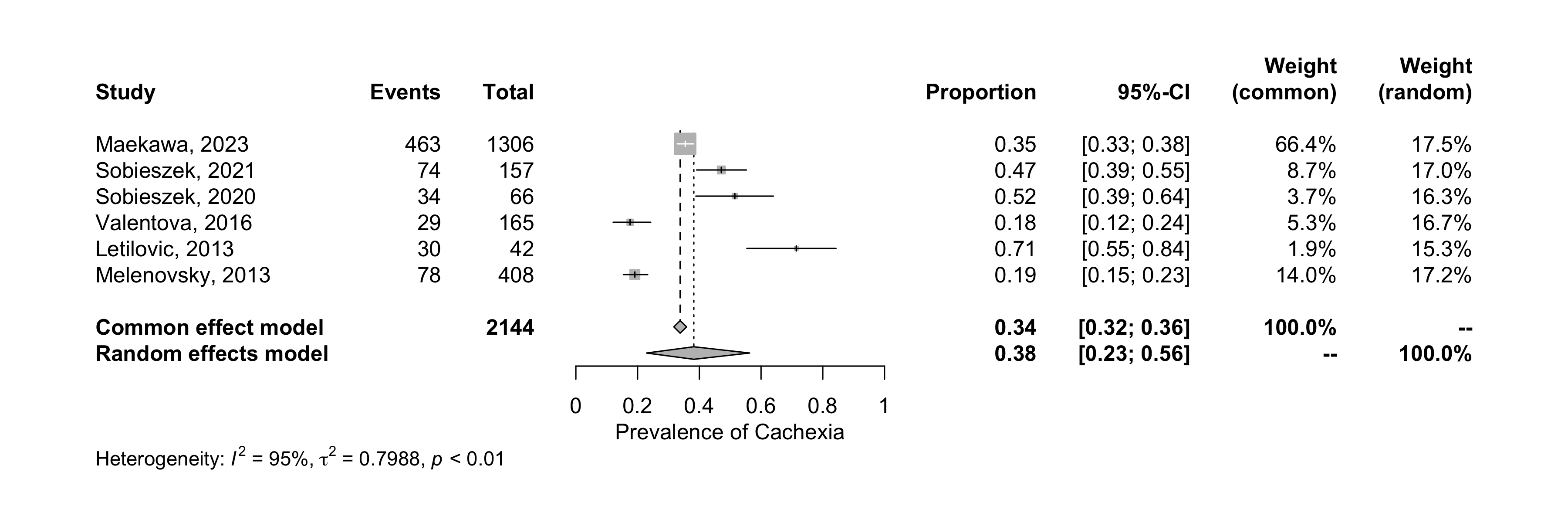

Supplement: Supplementary file 4 — Figure S4 Prevalence of cachexia defined by Evans’ criteria among patients with HF after exclusion of studies with high risk of bias [file JCSM-15-2536-s007.png]
