# Supplementary material for: Prognostic Impact and Prevalence of Cachexia in Patients With Heart Failure: A Systematic Review and Meta‐Analysis
Source: J Cachexia Sarcopenia Muscle. 2024 Oct 30;15(6):2536–43. doi: 10.1002/jcsm.13596 (PMC11634528; doi:10.1002/jcsm.13596)
Supplement: Supplementary file 5 — Table S1 Key terms employed in the screening of the literature search. [file JCSM-15-2536-s005.docx]

**Table S1.**Key terms employed in the screening of the literature search.

| **Database** | **Search terms** |
| --- | --- |
|  |  |
| PubMed | 1. "heart failure" AND "cache*" 2. ((("Heart Failure"[MeSH Terms] OR ("cardiac failure"[Title/Abstract] OR "heart decompensation"[Title/Abstract]   OR "coronary infarction"[Title/Abstract] OR "ventricular dysfunction"[Title/Abstract]  OR "cardiac infarction"[Title/Abstract] OR "congestive heart failure"[Title/Abstract]  OR "heart failure"[Title/Abstract])) AND ((cachexia[MeSH Terms]) OR "cachexia"[Title/Abstract]  OR "muscle wasting"[Title/Abstract] OR "muscle loss"[Title/Abstract] OR "cardiac cachexia"[Title/Abstract]  OR "cache*"[Title/Abstract])) |
| Cochrane Library | 1. "heart failure" AND "cache*" 2. ("heart failure" OR "cardiac failure" OR "heart decompensation" OR "coronary infarction" OR "ventricular dysfunction"   OR "cardiac infarction" OR "congestive heart failure")  AND ("cachexia" OR "muscle wasting" OR "muscle loss" OR "cardiac cachexia" OR "cache*") |
| Web of Science | 1. "heart failure" AND "cache*" 2. TS=("heart failure" OR "cardiac failure" OR "heart decompensation" OR "coronary infarction"   OR "ventricular dysfunction" OR "cardiac infarction" OR "congestive heart failure"))  AND (TS=("cachexia" OR "muscle wasting" OR "muscle loss" OR "cardiac cachexia" OR "cache*") |
| Scopus | 1. "heart failure" AND "cache*" 2. TITLE-ABS-KEY(("heart failure" OR "cardiac failure" OR "heart decompensation" OR   "coronary infarction" OR "ventricular dysfunction" OR "cardiac infarction" OR "congestive heart failure") AND  ("cachexia" OR "muscle wasting" OR "muscle loss" OR "cardiac cachexia" OR "cache*")) |
