# Supplementary material for: Prognostic Impact and Prevalence of Cachexia in Patients With Heart Failure: A Systematic Review and Meta‐Analysis
Source: J Cachexia Sarcopenia Muscle. 2024 Oct 30;15(6):2536–43. doi: 10.1002/jcsm.13596 (PMC11634528; doi:10.1002/jcsm.13596)
Supplement: Supplementary file 6 — Table S2 PICO characteristics of research questions in this systematic review. [file JCSM-15-2536-s013.docx]

**Table S2.** PICO characteristics of research questions in this systematic review.

| **Questions** | **Patient population** | **Exposure** | **Comparison** | **Outcome** |
| --- | --- | --- | --- | --- |
| Do patients with HF and diagnosed with cachexia have higher mortality than patients without cachexia? | Patients with heart failure over 18 years old irrespective of clinical settings | Cachexia diagnosed based on Evans’ criteria | No cachexia based on Evans’ criteria | All-cause mortality |
| What is the risk of cachexia among patients with HF? | Patients with heart failure over 18 years old irrespective of clinical settings | - | - | Cachexia diagnosed based on Evans’ criteria |

HF, heart failure
