# Supplementary material for: Prognostic Impact and Prevalence of Cachexia in Patients With Heart Failure: A Systematic Review and Meta‐Analysis
Source: J Cachexia Sarcopenia Muscle. 2024 Oct 30;15(6):2536–43. doi: 10.1002/jcsm.13596 (PMC11634528; doi:10.1002/jcsm.13596)
Supplement: Supplementary file 7 — Table S3 Study outcomes and variables adjusted for in the multivariable analyses. [file JCSM-15-2536-s002.docx]

**Table S3.** Study outcomes and variables adjusted for in the multivariable analyses.

| **Study, Year** | **Mortality adjusted for confounders, HR (95% CI)** | **Confounders adjusted for** |
| --- | --- | --- |
| Maekawa et al., 2023 | 1.49 (1.17-1.90) | BNP and MAGGIC score comprising: age, sex, LVEF, BMI, creatinine level, NYHA class, smoking status, complications (diabetes and chronic obstructive pulmonary disease), history of HF, and medication use (ACE inhibitors, ARBs, and beta-blockers) |
| Sobieszek et al., 2021 | - | no adjustment was made |
| Morishita et al., 2021 | 1.89 (1.06-3.37) | age, sex, eGFR and LVEF |
| Saitoh et al., 2017 | 2.99 (1.00 - 9.00) | age, gender, LVEF, and NYHA class |
| Szabo et al., 2014 | - | no adjustment was made |
| Melenovsky et al., 2013 | 1.68 (1.02 - 2.70) | age, heart rate, RVD, ACE inhibitors or ARBs use, tricuspid regurgitation gradient and BNP |
