# Supplementary material for: Prognostic Impact and Prevalence of Cachexia in Patients With Heart Failure: A Systematic Review and Meta‐Analysis
Source: J Cachexia Sarcopenia Muscle. 2024 Oct 30;15(6):2536–43. doi: 10.1002/jcsm.13596 (PMC11634528; doi:10.1002/jcsm.13596)
Supplement: Supplementary file 8 — Table S4 Meta‐regression analyses of patients with heart failure and cachexia vs. patients with heart failure without cachexia using Evans’ criteria. [file JCSM-15-2536-s010.docx]

**Table S4.** Meta-regression analyses of patients with heart failure and cachexia vs. patients with heart failure without cachexia using Evans’ criteria.

| **Confounder** | *r* | SE | 95%CI | *z* | P |
| --- | --- | --- | --- | --- | --- |
| Age | 0.0253 | 0.058 | -0.09 – 0.14 | 0.43 | 0.66 |
| BMI | 0.2290 | 0.122 | -0.01 – 0.47 | 1.88 | 0.06 |

Abbreviations: BMI - body mass index
