# Supplementary material for: Prognostic Impact and Prevalence of Cachexia in Patients With Heart Failure: A Systematic Review and Meta‐Analysis
Source: J Cachexia Sarcopenia Muscle. 2024 Oct 30;15(6):2536–43. doi: 10.1002/jcsm.13596 (PMC11634528; doi:10.1002/jcsm.13596)
Supplement: Supplementary file 9 — Table S5 Risk of bias assessment for studies evaluating impact of cachexia on mortality. [file JCSM-15-2536-s001.docx]

**Table S5.** Risk of bias assessment for studies evaluating impact of cachexia on mortality.

| **Study, year** | **Q1** | **Q2** | **Q3** | **Q4** | **Q5** | **Q6** | **Q7** | **Q8** | **Q9** | **Q10** | **Q11** | **Q12** | **Q13** | **Q14** | **Overall** |
| --- | --- | --- | --- | --- | --- | --- | --- | --- | --- | --- | --- | --- | --- | --- | --- |
| Maekawa et al. 2023 | Y | Y | NR | N | N | Y | Y | N | Y | N | Y | CD | N | Y | Good |
| Sobieszek et al. 2021 | N | Y | NR | N | N | Y | Y | Y | CD | N | N | CD | NR | N | Poor |
| Morishita et al. 2020 | Y | Y | NR | Y | N | Y | Y | N | N | N | Y | N | NR | Y | Poor |
| Saitoh et al. 2017 | Y | Y | NR | Y | N | Y | Y | Y | Y | N | N | CD | NR | Y | Fair |
| Szabo et al. 2014 | Y | N | NR | CD | N | Y | Y | N | Y | N | N | CD | NR | N | Poor |
| Melenovsky et al. 2013 | N | Y | NR | Y | N | Y | Y | N | Y | N | CD | CD | NR | Y | Fair |

## Abbreviations: Y - Yes, N - No, NR - Not Reported, CD - Cannot Determine.

## 01. Maekawa et al. 2023 (FRAGILE-HF)

|  | Yes | No | Other (CD, NR, NA) |
| --- | --- | --- | --- |
| 1. Was the research question or objective in this paper clearly stated? | Y |  |  |
| 2. Was the study population clearly specified and defined? | Y |  |  |
| 3. Was the participation rate of eligible persons at least 50%? |  |  | NR |
| 4. Were all the subjects selected or recruited from the same or similar populations (including the same time period)? Were inclusion and exclusion criteria for being in the study prespecified and applied uniformly to all participants?  Assessment: Patients were excluded based on the natriuretic peptides levels, or if the was no data on natriuretic peptides. This may result in bias, as those without data may differ. |  | N |  |
| 5. Was a sample size justification, power description, or variance and effect estimates provided?  Assessment: No mention. |  | N |  |
| 6. For the analyses in this paper, were the exposure(s) of interest measured prior to the outcome(s) being measured? | Y |  |  |
| 7. Was the **timeframe** sufficient so that one could reasonably expect to see an association between exposure and outcome if it existed? | Y |  |  |
| 8. For exposures that can vary in amount or level, did the study examine different levels of the exposure as related to the outcome (e.g., categories of exposure, or exposure measured as continuous variable)?  Assessment: Cachexia as a binary variable |  | N |  |
| 9. Were the exposure measures (independent variables) clearly defined, valid, reliable, and implemented consistently across all study participants? | Y |  |  |
| 10. Was the exposure(s) assessed more than once over time?  Assessment: All tests prior to discharge, no mention of repeating the tests |  | N |  |
| 11. Were the outcome measures (dependent variables) clearly defined, valid, reliable, and implemented consistently across all study participants?  Assessment: All cause death, followed in clinic and via telephone interview, not ideal but satisfactory | Y |  |  |
| 12. Were the outcome assessors blinded to the exposure status of participants?  Assessment: No mention in the paper, technically they might not be blinded. |  |  | CD |
| 13. Was loss to follow-up after baseline 20% or less?  Assessment: A complete follow-up of 2 years was performed in 78.0% of the total cohort. |  | N |  |
| 14. Were key potential confounding variables measured and adjusted statistically for their impact on the relationship between exposure(s) and outcome(s)?  Assessment: Adjusted for MAGGIC risk score and BNP levels (MAGGIC consists of 13 variables). | Y |  |  |

*CD, cannot determine; NA, not applicable; NR, not reported

| Quality rating (Good/ Fair/ Poor) | Good |
| --- | --- |
| Rater 1 initials: | KI |
| Rater 2 initials: |  |
| Additional comments, if Poor – why? | The elements of the tool that were not accounted for by the authors do not influence the risk of bias substantially. |

## 02. Sobieszek et al. 2021

|  | Yes | No | Other (CD, NR, NA) |
| --- | --- | --- | --- |
| 1. Was the research question or objective in this paper clearly stated?  Assessment: “The aim of this study was to assess the potential role of sST2 and its potential utility in male patients with CHF under cachexia condition.” – utility for what? |  | N |  |
| 2. Was the study population clearly specified and defined? | Y |  |  |
| 3. Was the participation rate of eligible persons at least 50%? |  |  | NR |
| 4. Were all the subjects selected or recruited from the same or similar populations (including the same time period)? Were inclusion and exclusion criteria for being in the study prespecified and applied uniformly to all participants?  Assessment: Some patients were recruited from the clinics, some form cardiology ward. These population may differ in the rates of exposure. |  | N |  |
| 5. Was a sample size justification, power description, or variance and effect estimates provided?  Assessment: No mention. |  | N |  |
| 6. For the analyses in this paper, were the exposure(s) of interest measured prior to the outcome(s) being measured? | Y |  |  |
| 7. Was the **timeframe** sufficient so that one could reasonably expect to see an association between exposure and outcome if it existed? | Y |  |  |
| 8. For exposures that can vary in amount or level, did the study examine different levels of the exposure as related to the outcome (e.g., categories of exposure, or exposure measured as continuous variable)?  Assessment: Cachexia with the presence of some additional biomarkers. | Y |  |  |
| 9. Were the exposure measures (independent variables) clearly defined, valid, reliable, and implemented consistently across all study participants?  Assessment: The authors did not describe all of the methods used to ascertain cachexia (which are laborious and maybe not done consistently?). |  |  | CD |
| 10. Was the exposure(s) assessed more than once over time? |  | N |  |
| 11. Were the outcome measures (dependent variables) clearly defined, valid, reliable, and implemented consistently across all study participants?  Assessment: No mention of how death was ascertained |  | N |  |
| 12. Were the outcome assessors blinded to the exposure status of participants? |  |  | CD |
| 13. Was loss to follow-up after baseline 20% or less? |  |  | NR |
| 14. Were key potential confounding variables measured and adjusted statistically for their impact on the relationship between exposure(s) and outcome(s)?  Assessment: No multivariate model with cachexia included |  | N |  |

*CD, cannot determine; NA, not applicable; NR, not reported

| Quality rating (Good/ Fair/ Poor) | Poor |
| --- | --- |
| Rater 1 initials: | KI |
| Rater 2 initials: |  |
| Additional comments, if Poor – why? | The ascertainment of both cachexia and mortality is uncertain. The authors mention these very briefly and not comprehensively or do not mention it at all (outcome). 5-year follow up is long, yet no data on loss to follow up or even method of data collection were reported. No adjustment for confounding has been applied. |

## 03. Morishita et al. 2020

|  | Yes | No | Other (CD, NR, NA) |
| --- | --- | --- | --- |
| 1. Was the research question or objective in this paper clearly stated? | Y |  |  |
| 2. Was the study population clearly specified and defined? | Y |  |  |
| 3. Was the participation rate of eligible persons at least 50%? |  |  | NR |
| 4. Were all the subjects selected or recruited from the same or similar populations (including the same time period)? Were inclusion and exclusion criteria for being in the study prespecified and applied uniformly to all participants? | Y |  |  |
| 5. Was a sample size justification, power description, or variance and effect estimates provided?  Assessment: No mention. |  | N |  |
| 6. For the analyses in this paper, were the exposure(s) of interest measured prior to the outcome(s) being measured? | Y |  |  |
| 7. Was the **timeframe** sufficient so that one could reasonably expect to see an association between exposure and outcome if it existed? | Y |  |  |
| 8. For exposures that can vary in amount or level, did the study examine different levels of the exposure as related to the outcome (e.g., categories of exposure, or exposure measured as continuous variable)?  Assessment: Cachexia was treated as a binary variable. |  | N |  |
| 9. Were the exposure measures (independent variables) clearly defined, valid, reliable, and implemented consistently across all study participants?  Assessment: The cachexia was ascertained retrospectively: “For most heart failure patients, the medical records did not include a standardized cachexia questionnaire, such as decreased muscle strength (grip strength), low-fat mass index (assessed by bioelectrical impedance) and the measurement of interleukin-6.” This may result in bias. Elevated CRP or anaemia for example, may be the result of other acute illness. |  | N |  |
| 10. Was the exposure(s) assessed more than once over time? |  | N |  |
| 11. Were the outcome measures (dependent variables) clearly defined, valid, reliable, and implemented consistently across all study participants?  Assessment: All-cause death. | Y |  |  |
| 12. Were the outcome assessors blinded to the exposure status of participants?  Assessment: Both exposure and outcome assessed retrospectively. |  | N |  |
| 13. Was loss to follow-up after baseline 20% or less? |  |  | NR |
| 14. Were key potential confounding variables measured and adjusted statistically for their impact on the relationship between exposure(s) and outcome(s)?  Assessment: Age, sex, ejection fraction, eGFR were adjusted for. According to random forest modelling, all important predictors were adjusted for, except for variables that constitute cachexia. | Y |  |  |

*CD, cannot determine; NA, not applicable; NR, not reported

| Quality rating (Good/ Fair/ Poor) | Poor |
| --- | --- |
| Rater 1 initials: | KI |
| Rater 2 initials: |  |
| Additional comments, if Poor – why? | Cachexia was ascertained retrospectively based on the limited data, however, the decision was made by clinician upon reviewing the case. Outcome of interest was reviewed retrospectively as well and chart review for that could be not sufficient. |

## 04. Saitoh et al. 2017 (SICA-HF)

|  | Yes | No | Other (CD, NR, NA) |
| --- | --- | --- | --- |
| 1. Was the research question or objective in this paper clearly stated? | Y |  |  |
| 2. Was the study population clearly specified and defined? | Y |  |  |
| 3. Was the participation rate of eligible persons at least 50%? |  |  | NR |
| 4. Were all the subjects selected or recruited from the same or similar populations (including the same time period)? Were inclusion and exclusion criteria for being in the study prespecified and applied uniformly to all participants? | Y |  |  |
| 5. Was a sample size justification, power description, or variance and effect estimates provided?  Assessment: No mention. |  | N |  |
| 6. For the analyses in this paper, were the exposure(s) of interest measured prior to the outcome(s) being measured? | Y |  |  |
| 7. Was the **timeframe** sufficient so that one could reasonably expect to see an association between exposure and outcome if it existed? | Y |  |  |
| 8. For exposures that can vary in amount or level, did the study examine different levels of the exposure as related to the outcome (e.g., categories of exposure, or exposure measured as continuous variable)?  Assessment: Anorexia with and without cachexia | Y |  |  |
| 9. Were the exposure measures (independent variables) clearly defined, valid, reliable, and implemented consistently across all study participants? | Y |  |  |
| 10. Was the exposure(s) assessed more than once over time? |  | N |  |
| 11. Were the outcome measures (dependent variables) clearly defined, valid, reliable, and implemented consistently across all study participants?  Assessment: No information on how death was ascertained. |  | N |  |
| 12. Were the outcome assessors blinded to the exposure status of participants?  Assessment: No information on how death was ascertained. |  |  | CD |
| 13. Was loss to follow-up after baseline 20% or less? |  |  | NR |
| 14. Were key potential confounding variables measured and adjusted statistically for their impact on the relationship between exposure(s) and outcome(s)?  Assessment: Adjusted for age, gender, left ventricular ejection fraction, and New York Heart Association class | Y |  |  |

*CD, cannot determine; NA, not applicable; NR, not reported

| Quality rating (Good/ Fair/ Poor) | Fair |
| --- | --- |
| Rater 1 initials: | KI |
| Rater 2 initials: |  |
| Additional comments, if Poor – why? | The authors did not specify how the follow up data was collected and the rate of loss to follow up. Other than that the study seems well conducted. |

## 05. Szabo et al. 2014

|  | Yes | No | Other (CD, NR, NA) |
| --- | --- | --- | --- |
| 1. Was the research question or objective in this paper clearly stated? | Y |  |  |
| 2. Was the study population clearly specified and defined?  Assessment: No settings, exact location and timeframe specified. |  | N |  |
| 3. Was the participation rate of eligible persons at least 50%? |  |  | NR |
| 4. Were all the subjects selected or recruited from the same or similar populations (including the same time period)? Were inclusion and exclusion criteria for being in the study prespecified and applied uniformly to all participants?  Assessment: It is not reported where and when the participants were recruited, this is a potential risk of bias. |  |  | CD |
| 5. Was a sample size justification, power description, or variance and effect estimates provided?  Assessment: No mention. |  | N |  |
| 6. For the analyses in this paper, were the exposure(s) of interest measured prior to the outcome(s) being measured?  Assessment: Mortality | Y |  |  |
| 7. Was the **timeframe** sufficient so that one could reasonably expect to see an association between exposure and outcome if it existed? | Y |  |  |
| 8. For exposures that can vary in amount or level, did the study examine different levels of the exposure as related to the outcome (e.g., categories of exposure, or exposure measured as continuous variable)? |  | N |  |
| 9. Were the exposure measures (independent variables) clearly defined, valid, reliable, and implemented consistently across all study participants? | Y |  |  |
| 10. Was the exposure(s) assessed more than once over time? |  | N |  |
| 11. Were the outcome measures (dependent variables) clearly defined, valid, reliable, and implemented consistently across all study participants?  Assessment: No information on how mortality data were collected. |  | N |  |
| 12. Were the outcome assessors blinded to the exposure status of participants? |  |  | CD |
| 13. Was loss to follow-up after baseline 20% or less? |  |  | NR |
| 14. Were key potential confounding variables measured and adjusted statistically for their impact on the relationship between exposure(s) and outcome(s)?  Assessment: No multivariable analysis with cachexia |  | N |  |

*CD, cannot determine; NA, not applicable; NR, not reported

| Quality rating (Good/ Fair/ Poor) | Poor |
| --- | --- |
| Rater 1 initials: | KI |
| Rater 2 initials: |  |
| Additional comments, if Poor – why? | The authors did not report on how mortality data was collected, the paragraph regarding recruitment is rather vague, no settings, location, time and utilized HF definition are reported. No multivariate model with cachexia was developed. |

## 06. Melenovsky et al. 2013

|  | Yes | No | Other (CD, NR, NA) |
| --- | --- | --- | --- |
| 1. Was the research question or objective in this paper clearly stated?  Assessment: Vague hypothesis, no specific aim. |  | N |  |
| 2. Was the study population clearly specified and defined? | Y |  |  |
| 3. Was the participation rate of eligible persons at least 50%? |  |  | NR |
| 4. Were all the subjects selected or recruited from the same or similar populations (including the same time period)? Were inclusion and exclusion criteria for being in the study prespecified and applied uniformly to all participants? | Y |  |  |
| 5. Was a sample size justification, power description, or variance and effect estimates provided?  Assessment: No mention. |  | N |  |
| 6. For the analyses in this paper, were the exposure(s) of interest measured prior to the outcome(s) being measured? | Y |  |  |
| 7. Was the **timeframe** sufficient so that one could reasonably expect to see an association between exposure and outcome if it existed? | Y |  |  |
| 8. For exposures that can vary in amount or level, did the study examine different levels of the exposure as related to the outcome (e.g., categories of exposure, or exposure measured as continuous variable)? |  | N |  |
| 9. Were the exposure measures (independent variables) clearly defined, valid, reliable, and implemented consistently across all study participants? | Y |  |  |
| 10. Was the exposure(s) assessed more than once over time? |  | N |  |
| 11. Were the outcome measures (dependent variables) clearly defined, valid, reliable, and implemented consistently across all study participants?  Assessment: No information on how adverse events data were collected. |  |  | CD |
| 12. Were the outcome assessors blinded to the exposure status of participants? |  |  | CD |
| 13. Was loss to follow-up after baseline 20% or less? |  |  | NR |
| 14. Were key potential confounding variables measured and adjusted statistically for their impact on the relationship between exposure(s) and outcome(s)?  Assessment: Model was adjusted for significant predictors, i.e. natremia, age, gender, NYHA class, SBP, HR, IVC diameter, present RVD, TR gradient, ACEI or ARB use, BNP, LVEF, GFR, present leg edema. | Y |  |  |

*CD, cannot determine; NA, not applicable; NR, not reported

| Quality rating (Good/ Fair/ Poor) | Fair |
| --- | --- |
| Rater 1 initials: | KI |
| Rater 2 initials: |  |
| Additional comments, if Poor – why? | The study did not specify how adverse outcomes were ascertained. Precise description of how criteria for cachexia were measured. Overall low risk of bias. |
