# Supplementary material for: Prognostic Impact and Prevalence of Cachexia in Patients With Heart Failure: A Systematic Review and Meta‐Analysis
Source: J Cachexia Sarcopenia Muscle. 2024 Oct 30;15(6):2536–43. doi: 10.1002/jcsm.13596 (PMC11634528; doi:10.1002/jcsm.13596)
Supplement: Supplementary file 10 — Table S6 Risk of bias assessment for studies utilized to assess prevalence of cachexia using Evans criteria. [file JCSM-15-2536-s011.docx]

**Table S6.** Risk of bias assessment for studies utilized to assess prevalence of cachexia using Evans criteria.

| **Study, year** | **Q1** | **Q2** | **Q3** | **Q4** | **Q5** | **Q6** | **Q7** | **Q8** | **Q9** | **Q10** | **Q11** | **Q12** | **Q13** | **Q14** | **Overall Rating** |
| --- | --- | --- | --- | --- | --- | --- | --- | --- | --- | --- | --- | --- | --- | --- | --- |
| Armas et al. 2023 | N | N | Y | CD | N | Y | N | NA | Y | NA | N | N | NR | N | Poor |
| Carson et al. 2022 | Y | Y | CD | N | Y | N | N | N | Y | N | Y | N | NA | N | Poor |
| Letilovic et al. 2013 | Y | Y | Y | Y | N | Y | Y | Y | Y | N | Y | CD | Y | N | Good |
| Maekawa et al. 2023 | Y | Y | NR | N | N | Y | Y | N | Y | N | Y | CD | N | Y | Good |
| Melenovsky et al. 2013 | N | Y | NR | Y | N | Y | Y | N | Y | N | CD | CD | NR | Y | Fair |
| Morishita et al. 2020 | Y | Y | NR | Y | N | Y | Y | N | N | N | Y | N | NR | Y | Poor |
| Sobieszek et al. 2020 | Y | Y | NR | Y | N | N | N | N | Y | N | Y | N | NA | N | Fair |
| Sobieszek et al. 2021 | Y | Y | NR | Y | N | N | N | N | Y | N | Y | N | NA | N | Fair |
| Szabo et al. 2014 | Y | N | NR | CD | N | Y | Y | N | Y | N | N | CD | NR | N | Poor |
| Valentova et al. 2016 | Y | Y | Y | Y | N | N | N | N | Y | N | Y | Y | NA | Y | Good |

## Abbreviations: Y - Yes, N - No, NR - Not reported, CD - Cannot determine, NA – Not applicable

## 01. Armas et al. 2023

|  | Yes | No | Other (CD, NR, NA) |
| --- | --- | --- | --- |
| 1. Was the research question or objective in this paper clearly stated?  Assessment: No, the authors give vague description of what they’ve done and state their conclusions in the introduction. |  | N |  |
| 2. Was the study population clearly specified and defined?  Assessment: No data on the centre, hospital or ward that the patients were recruited from. “Records from January 2017 to January 2022 were searched. All patients who received support from an Impella 5.0 or 5.5 before implantation of a D-LVAD were screened.” |  | N |  |
| 3. Was the participation rate of eligible persons at least 50%?  Assessment: All patients with Impella support and D-LVAD implantation were screened and included. | Y |  |  |
| 4. Were all the subjects selected or recruited from the same or similar populations (including the same time period)? Were inclusion and exclusion criteria for being in the study prespecified and applied uniformly to all participants?  Assessment: The recruitment site was not specified, not possible to discern whether cachectic were included from the same population. |  |  | CD |
| 5. Was a sample size justification, power description, or variance and effect estimates provided?  Assessment: No |  | N |  |
| 6. For the analyses in this paper, were the exposure(s) of interest measured prior to the outcome(s) being measured?  Assessment: Assuming cachexia is an exposure. Data related to cachexia collected during hospitalization, then outcome is 30-day survival. | Y |  |  |
| 7. Was the **timeframe** sufficient so that one could reasonably expect to see an association between exposure and outcome if it existed?  Assessment: 100% survival at 30 days and 6 months |  | N |  |
| 8. For exposures that can vary in amount or level, did the study examine different levels of the exposure as related to the outcome (e.g., categories of exposure, or exposure measured as continuous variable)?  Assessment: Outcomes not compared according to cachexia. |  |  | NA |
| 9. Were the exposure measures (independent variables) clearly defined, valid, reliable, and implemented consistently across all study participants?  Assessment: Clearly stated definition of cachexia in Table 1. | Y |  |  |
| 10. Was the exposure(s) assessed more than once over time? |  |  | NA |
| 11. Were the outcome measures (dependent variables) clearly defined, valid, reliable, and implemented consistently across all study participants?  Assessment: The authors did not state how the survival was assessed. |  | N |  |
| 12. Were the outcome assessors blinded to the exposure status of participants?  Assessment: Study is a case series |  | N |  |
| 13. Was loss to follow-up after baseline 20% or less?  Assessment: |  |  | NR |
| 14. Were key potential confounding variables measured and adjusted statistically for their impact on the relationship between exposure(s) and outcome(s)?  Assessment: |  | N |  |

*CD, cannot determine; NA, not applicable; NR, not reported

| Quality rating (Good/ Fair/ Poor) | Poor |
| --- | --- |
| Rater 1 initials: |  |
| Rater 2 initials: |  |
| Additional comments, if Poor – why? | This was a case series study without clearly specified aims or studied population. It remains unknown how the survival was assessed and this outcome did not allow for any meaningful difference to be shown (timeframe too short). |

## 02. Carson et al. 2022

|  | Yes | No | Other (CD, NR, NA) |
| --- | --- | --- | --- |
| 1. Was the research question or objective in this paper clearly stated?  Assessment: Only partly true. The secondary objective is vague: ‘explore its impact on patients and caregivers’. | Y |  |  |
| 2. Was the study population clearly specified and defined?  Assessment: | Y |  |  |
| 3. Was the participation rate of eligible persons at least 50%?  Assessment: The screening process not clearly explained. |  |  | CD |
| 4. Were all the subjects selected or recruited from the same or similar populations (including the same time period)? Were inclusion and exclusion criteria for being in the study prespecified and applied uniformly to all participants?  Assessment: Recruitment of both inpatients and outpatients, no data on the rate of cachexia in these different populations. |  | N |  |
| 5. Was a sample size justification, power description, or variance and effect estimates provided?  Assessment: | Y |  |  |
| 6. For the analyses in this paper, were the exposure(s) of interest measured prior to the outcome(s) being measured?  Assessment: Cross-sectional study |  | N |  |
| 7. Was the **timeframe** sufficient so that one could reasonably expect to see an association between exposure and outcome if it existed?  Assessment: Cross-sectional study |  | N |  |
| 8. For exposures that can vary in amount or level, did the study examine different levels of the exposure as related to the outcome (e.g., categories of exposure, or exposure measured as continuous variable)?  Assessment: The cachexia is treated only as a binary variable |  | N |  |
| 9. Were the exposure measures (independent variables) clearly defined, valid, reliable, and implemented consistently across all study participants?  Assessment: Direct measurements and validated questionnaires | Y |  |  |
| 10. Was the exposure(s) assessed more than once over time?  Assessment: |  | N |  |
| 11. Were the outcome measures (dependent variables) clearly defined, valid, reliable, and implemented consistently across all study participants?  Assessment: | Y |  |  |
| 12. Were the outcome assessors blinded to the exposure status of participants?  Assessment: Cross-sectional sequential study |  | N |  |
| 13. Was loss to follow-up after baseline 20% or less?  Assessment: |  |  | NA |
| 14. Were key potential confounding variables measured and adjusted statistically for their impact on the relationship between exposure(s) and outcome(s)?  Assessment: |  | N |  |

*CD, cannot determine; NA, not applicable; NR, not reported

| Quality rating (Good/ Fair/ Poor) | Poor |
| --- | --- |
| Rater 1 initials: | KI |
| Rater 2 initials: |  |
| Additional comments, if Poor – why? | This was a cross-sectional study that aimed to evaluate prevalence of cachexia in HF and recruited patients in different settings, but there is no stratification by the settings. The other aims are vague and exploratory. No effect estimates are reported and there is no adjustment for confounding. |

## 03. Letilovic 2013 et al.

|  | Yes | No | Other (CD, NR, NA) |
| --- | --- | --- | --- |
| 1. Was the research question or objective in this paper clearly stated?  Assessment: | Y |  |  |
| 2. Was the study population clearly specified and defined?  Assessment: | Y |  |  |
| 3. Was the participation rate of eligible persons at least 50%?  Assessment: | Y |  |  |
| 4. Were all the subjects selected or recruited from the same or similar populations (including the same time period)? Were inclusion and exclusion criteria for being in the study prespecified and applied uniformly to all participants?  Assessment: | Y |  |  |
| 5. Was a sample size justification, power description, or variance and effect estimates provided?  Assessment: |  | N |  |
| 6. For the analyses in this paper, were the exposure(s) of interest measured prior to the outcome(s) being measured?  Assessment: | Y |  |  |
| 7. Was the **timeframe** sufficient so that one could reasonably expect to see an association between exposure and outcome if it existed?  Assessment: | Y |  |  |
| 8. For exposures that can vary in amount or level, did the study examine different levels of the exposure as related to the outcome (e.g., categories of exposure, or exposure measured as continuous variable)?  Assessment: | Y |  |  |
| 9. Were the exposure measures (independent variables) clearly defined, valid, reliable, and implemented consistently across all study participants?  Assessment: | Y |  |  |
| 10. Was the exposure(s) assessed more than once over time?  Assessment: |  | N |  |
| 11. Were the outcome measures (dependent variables) clearly defined, valid, reliable, and implemented consistently across all study participants?  Assessment: | Y |  |  |
| 12. Were the outcome assessors blinded to the exposure status of participants?  Assessment: |  |  | CD |
| 13. Was loss to follow-up after baseline 20% or less?  Assessment: Probably, mortality assessment through National Registry of Deceased Persons. | Y |  |  |
| 14. Were key potential confounding variables measured and adjusted statistically for their impact on the relationship between exposure(s) and outcome(s)?  Assessment: |  | N |  |

*CD, cannot determine; NA, not applicable; NR, not reported

| Quality rating (Good/ Fair/ Poor) | Good |
| --- | --- |
| Rater 1 initials: | KI |
| Rater 2 initials: |  |
| Additional comments, if Poor – why? | Malignancy as an inclusion criterion. |

## 04. Maekawa et al. 2023 (FRAGILE-HF)

|  | Yes | No | Other (CD, NR, NA) |
| --- | --- | --- | --- |
| 1. Was the research question or objective in this paper clearly stated? | Y |  |  |
| 2. Was the study population clearly specified and defined? | Y |  |  |
| 3. Was the participation rate of eligible persons at least 50%? |  |  | NR |
| 4. Were all the subjects selected or recruited from the same or similar populations (including the same time period)? Were inclusion and exclusion criteria for being in the study prespecified and applied uniformly to all participants?  Assessment: Patients were excluded based on the natriuretic peptides levels, or if the was no data on natriuretic peptides. This may result in bias, as those without data may differ. |  | N |  |
| 5. Was a sample size justification, power description, or variance and effect estimates provided?  Assessment: No mention. |  | N |  |
| 6. For the analyses in this paper, were the exposure(s) of interest measured prior to the outcome(s) being measured? | Y |  |  |
| 7. Was the **timeframe** sufficient so that one could reasonably expect to see an association between exposure and outcome if it existed? | Y |  |  |
| 8. For exposures that can vary in amount or level, did the study examine different levels of the exposure as related to the outcome (e.g., categories of exposure, or exposure measured as continuous variable)?  Assessment: Cachexia as a binary variable |  | N |  |
| 9. Were the exposure measures (independent variables) clearly defined, valid, reliable, and implemented consistently across all study participants? | Y |  |  |
| 10. Was the exposure(s) assessed more than once over time?  Assessment: All tests prior to discharge, no mention of repeating the tests |  | N |  |
| 11. Were the outcome measures (dependent variables) clearly defined, valid, reliable, and implemented consistently across all study participants?  Assessment: All cause death, followed in clinic and via telephone interview, not ideal but satisfactory | Y |  |  |
| 12. Were the outcome assessors blinded to the exposure status of participants?  Assessment: No mention in the paper, technically they might not be blinded. |  |  | CD |
| 13. Was loss to follow-up after baseline 20% or less?  Assessment: A complete follow-up of 2 years was performed in 78.0% of the total cohort. |  | N |  |
| 14. Were key potential confounding variables measured and adjusted statistically for their impact on the relationship between exposure(s) and outcome(s)?  Assessment: Adjusted for MAGGIC risk score and BNP levels (MAGGIC consists of 13 variables). | Y |  |  |

*CD, cannot determine; NA, not applicable; NR, not reported

| Quality rating (Good/ Fair/ Poor) | Good |
| --- | --- |
| Rater 1 initials: | KI |
| Rater 2 initials: |  |
| Additional comments, if Poor – why? | The elements of the tool that were not accounted for by the authors do not influence the risk of bias substantially. |

## 05. Melenovsky et al. 2013

|  | Yes | No | Other (CD, NR, NA) |
| --- | --- | --- | --- |
| 1. Was the research question or objective in this paper clearly stated?  Assessment: Vague hypothesis, no specific aim. |  | N |  |
| 2. Was the study population clearly specified and defined? | Y |  |  |
| 3. Was the participation rate of eligible persons at least 50%? |  |  | NR |
| 4. Were all the subjects selected or recruited from the same or similar populations (including the same time period)? Were inclusion and exclusion criteria for being in the study prespecified and applied uniformly to all participants? | Y |  |  |
| 5. Was a sample size justification, power description, or variance and effect estimates provided?  Assessment: No mention. |  | N |  |
| 6. For the analyses in this paper, were the exposure(s) of interest measured prior to the outcome(s) being measured? | Y |  |  |
| 7. Was the **timeframe** sufficient so that one could reasonably expect to see an association between exposure and outcome if it existed? | Y |  |  |
| 8. For exposures that can vary in amount or level, did the study examine different levels of the exposure as related to the outcome (e.g., categories of exposure, or exposure measured as continuous variable)? |  | N |  |
| 9. Were the exposure measures (independent variables) clearly defined, valid, reliable, and implemented consistently across all study participants? | Y |  |  |
| 10. Was the exposure(s) assessed more than once over time? |  | N |  |
| 11. Were the outcome measures (dependent variables) clearly defined, valid, reliable, and implemented consistently across all study participants?  Assessment: No information on how adverse events data were collected. |  |  | CD |
| 12. Were the outcome assessors blinded to the exposure status of participants? |  |  | CD |
| 13. Was loss to follow-up after baseline 20% or less? |  |  | NR |
| 14. Were key potential confounding variables measured and adjusted statistically for their impact on the relationship between exposure(s) and outcome(s)?  Assessment: Model was adjusted for significant predictors, i.e. natremia, age, gender, NYHA class, SBP, HR, IVC diameter, present RVD, TR gradient, ACEI or ARB use, BNP, LVEF, GFR, present leg edema. | Y |  |  |

*CD, cannot determine; NA, not applicable; NR, not reported

| Quality rating (Good/ Fair/ Poor) | Fair |
| --- | --- |
| Rater 1 initials: | KI |
| Rater 2 initials: |  |
| Additional comments, if Poor – why? | The study did not specify how adverse outcomes were ascertained. Precise description of how criteria for cachexia were measured. Overall low risk of bias. |

## 06. Morishita et al. 2020

|  | Yes | No | Other (CD, NR, NA) |
| --- | --- | --- | --- |
| 1. Was the research question or objective in this paper clearly stated? | Y |  |  |
| 2. Was the study population clearly specified and defined? | Y |  |  |
| 3. Was the participation rate of eligible persons at least 50%? |  |  | NR |
| 4. Were all the subjects selected or recruited from the same or similar populations (including the same time period)? Were inclusion and exclusion criteria for being in the study prespecified and applied uniformly to all participants? | Y |  |  |
| 5. Was a sample size justification, power description, or variance and effect estimates provided?  Assessment: No mention. |  | N |  |
| 6. For the analyses in this paper, were the exposure(s) of interest measured prior to the outcome(s) being measured? | Y |  |  |
| 7. Was the **timeframe** sufficient so that one could reasonably expect to see an association between exposure and outcome if it existed? | Y |  |  |
| 8. For exposures that can vary in amount or level, did the study examine different levels of the exposure as related to the outcome (e.g., categories of exposure, or exposure measured as continuous variable)?  Assessment: Cachexia was treated as a binary variable. |  | N |  |
| 9. Were the exposure measures (independent variables) clearly defined, valid, reliable, and implemented consistently across all study participants?  Assessment: The cachexia was ascertained retrospectively: “For most heart failure patients, the medical records did not include a standardized cachexia questionnaire, such as decreased muscle strength (grip strength), low-fat mass index (assessed by bioelectrical impedance) and the measurement of interleukin-6.” This may result in bias. Elevated CRP or anaemia for example, may be the result of other acute illness. |  | N |  |
| 10. Was the exposure(s) assessed more than once over time? |  | N |  |
| 11. Were the outcome measures (dependent variables) clearly defined, valid, reliable, and implemented consistently across all study participants?  Assessment: All-cause death. | Y |  |  |
| 12. Were the outcome assessors blinded to the exposure status of participants?  Assessment: Both exposure and outcome assessed retrospectively. |  | N |  |
| 13. Was loss to follow-up after baseline 20% or less? |  |  | NR |
| 14. Were key potential confounding variables measured and adjusted statistically for their impact on the relationship between exposure(s) and outcome(s)?  Assessment: Age, sex, ejection fraction, eGFR were adjusted for. According to random forest modelling, all important predictors were adjusted for, except for variables that constitute cachexia. | Y |  |  |

*CD, cannot determine; NA, not applicable; NR, not reported

| Quality rating (Good/ Fair/ Poor) | Poor |
| --- | --- |
| Rater 1 initials: | KI |
| Rater 2 initials: |  |
| Additional comments, if Poor – why? | Cachexia was ascertained retrospectively based on the limited data, however, the decision was made by clinician upon reviewing the case. Outcome of interest was reviewed retrospectively as well and chart review for that could be not sufficient. |

## 07. Sobieszek et al. 2020

|  | Yes | No | Other (CD, NR, NA) |
| --- | --- | --- | --- |
| 1. Was the research question or objective in this paper clearly stated? | Y |  |  |
| 2. Was the study population clearly specified and defined? | Y |  |  |
| 3. Was the participation rate of eligible persons at least 50%? |  |  | NR |
| 4. Were all the subjects selected or recruited from the same or similar populations (including the same time period)? Were inclusion and exclusion criteria for being in the study prespecified and applied uniformly to all participants? | Y |  |  |
| 5. Was a sample size justification, power description, or variance and effect estimates provided? |  | N |  |
| 6. For the analyses in this paper, were the exposure(s) of interest measured prior to the outcome(s) being measured? |  | N |  |
| 7. Was the **timeframe** sufficient so that one could reasonably expect to see an association between exposure and outcome if it existed?  Assessment: Cross-sectional study. |  | N |  |
| 8. For exposures that can vary in amount or level, did the study examine different levels of the exposure as related to the outcome (e.g., categories of exposure, or exposure measured as continuous variable)? |  | N |  |
| 9. Were the exposure measures (independent variables) clearly defined, valid, reliable, and implemented consistently across all study participants? | Y |  |  |
| 10. Was the exposure(s) assessed more than once over time? |  | N |  |
| 11. Were the outcome measures (dependent variables) clearly defined, valid, reliable, and implemented consistently across all study participants? | Y |  |  |
| 12. Were the outcome assessors blinded to the exposure status of participants? |  | N |  |
| 13. Was loss to follow-up after baseline 20% or less? |  |  | NA |
| 14. Were key potential confounding variables measured and adjusted statistically for their impact on the relationship between exposure(s) and outcome(s)? | Y |  |  |

*CD, cannot determine; NA, not applicable; NR, not reported

| Quality rating (Good/ Fair/ Poor) | Fair |
| --- | --- |
| Rater 1 initials: | KI |
| Rater 2 initials: |  |
| Additional comments, if Poor – why? |  |

## 08. Sobieszek et al. 2021

|  | Yes | No | Other (CD, NR, NA) |
| --- | --- | --- | --- |
| 1. Was the research question or objective in this paper clearly stated? | Y |  |  |
| 2. Was the study population clearly specified and defined? | Y |  |  |
| 3. Was the participation rate of eligible persons at least 50%? |  |  | NR |
| 4. Were all the subjects selected or recruited from the same or similar populations (including the same time period)? Were inclusion and exclusion criteria for being in the study prespecified and applied uniformly to all participants? | Y |  |  |
| 5. Was a sample size justification, power description, or variance and effect estimates provided? |  | N |  |
| 6. For the analyses in this paper, were the exposure(s) of interest measured prior to the outcome(s) being measured? |  | N |  |
| 7. Was the **timeframe** sufficient so that one could reasonably expect to see an association between exposure and outcome if it existed?  Assessment: Cross-sectional study. |  | N |  |
| 8. For exposures that can vary in amount or level, did the study examine different levels of the exposure as related to the outcome (e.g., categories of exposure, or exposure measured as continuous variable)? |  | N |  |
| 9. Were the exposure measures (independent variables) clearly defined, valid, reliable, and implemented consistently across all study participants? | Y |  |  |
| 10. Was the exposure(s) assessed more than once over time? |  | N |  |
| 11. Were the outcome measures (dependent variables) clearly defined, valid, reliable, and implemented consistently across all study participants? | Y |  |  |
| 12. Were the outcome assessors blinded to the exposure status of participants?  Assessment: Likely not due to cross-sectional design. |  | N |  |
| 13. Was loss to follow-up after baseline 20% or less? |  |  | NA |
| 14. Were key potential confounding variables measured and adjusted statistically for their impact on the relationship between exposure(s) and outcome(s)? | Y |  |  |

*CD, cannot determine; NA, not applicable; NR, not reported

| Quality rating (Good/ Fair/ Poor) | Fair |
| --- | --- |
| Rater 1 initials: | KI |
| Rater 2 initials: |  |
| Additional comments, if Poor – why? |  |

## 09. Szabo et al. 2014

|  | Yes | No | Other (CD, NR, NA) |
| --- | --- | --- | --- |
| 1. Was the research question or objective in this paper clearly stated? | Y |  |  |
| 2. Was the study population clearly specified and defined?  Assessment: No settings, exact location and timeframe specified. |  | N |  |
| 3. Was the participation rate of eligible persons at least 50%? |  |  | NR |
| 4. Were all the subjects selected or recruited from the same or similar populations (including the same time period)? Were inclusion and exclusion criteria for being in the study prespecified and applied uniformly to all participants?  Assessment: It is not reported where and when the participants were recruited, this is a potential risk of bias. |  |  | CD |
| 5. Was a sample size justification, power description, or variance and effect estimates provided?  Assessment: No mention. |  | N |  |
| 6. For the analyses in this paper, were the exposure(s) of interest measured prior to the outcome(s) being measured?  Assessment: Mortality | Y |  |  |
| 7. Was the **timeframe** sufficient so that one could reasonably expect to see an association between exposure and outcome if it existed? | Y |  |  |
| 8. For exposures that can vary in amount or level, did the study examine different levels of the exposure as related to the outcome (e.g., categories of exposure, or exposure measured as continuous variable)? |  | N |  |
| 9. Were the exposure measures (independent variables) clearly defined, valid, reliable, and implemented consistently across all study participants? | Y |  |  |
| 10. Was the exposure(s) assessed more than once over time? |  | N |  |
| 11. Were the outcome measures (dependent variables) clearly defined, valid, reliable, and implemented consistently across all study participants?  Assessment: No information on how mortality data were collected. |  | N |  |
| 12. Were the outcome assessors blinded to the exposure status of participants? |  |  | CD |
| 13. Was loss to follow-up after baseline 20% or less? |  |  | NR |
| 14. Were key potential confounding variables measured and adjusted statistically for their impact on the relationship between exposure(s) and outcome(s)?  Assessment: No multivariable analysis with cachexia |  | N |  |

*CD, cannot determine; NA, not applicable; NR, not reported

| Quality rating (Good/ Fair/ Poor) | Poor |
| --- | --- |
| Rater 1 initials: | KI |
| Rater 2 initials: |  |
| Additional comments, if Poor – why? | The authors did not report on how mortality data was collected, the paragraph regarding recruitment is rather vague, no settings, location, time and utilized HF definition are reported. No multivariate model with cachexia was developed. |

## 10. Valentova et al. 2016

|  | Yes | No | Other (CD, NR, NA) |
| --- | --- | --- | --- |
| 1. Was the research question or objective in this paper clearly stated? | Y |  |  |
| 2. Was the study population clearly specified and defined? | Y |  |  |
| 3. Was the participation rate of eligible persons at least 50%? | Y |  |  |
| 4. Were all the subjects selected or recruited from the same or similar populations (including the same time period)? Were inclusion and exclusion criteria for being in the study prespecified and applied uniformly to all participants? | Y |  |  |
| 5. Was a sample size justification, power description, or variance and effect estimates provided? |  | N |  |
| 6. For the analyses in this paper, were the exposure(s) of interest measured prior to the outcome(s) being measured?  Assessment: Cross-sectional study. |  | N |  |
| 7. Was the **timeframe** sufficient so that one could reasonably expect to see an association between exposure and outcome if it existed? |  | N |  |
| 8. For exposures that can vary in amount or level, did the study examine different levels of the exposure as related to the outcome (e.g., categories of exposure, or exposure measured as continuous variable)? |  | N |  |
| 9. Were the exposure measures (independent variables) clearly defined, valid, reliable, and implemented consistently across all study participants? | Y |  |  |
| 10. Was the exposure(s) assessed more than once over time? |  | N |  |
| 11. Were the outcome measures (dependent variables) clearly defined, valid, reliable, and implemented consistently across all study participants? | Y |  |  |
| 12. Were the outcome assessors blinded to the exposure status of participants? | Y |  |  |
| 13. Was loss to follow-up after baseline 20% or less? |  |  | NA |
| 14. Were key potential confounding variables measured and adjusted statistically for their impact on the relationship between exposure(s) and outcome(s)? | Y |  |  |

*CD, cannot determine; NA, not applicable; NR, not reported

| Quality rating (Good/ Fair/ Poor) | Good |
| --- | --- |
| Rater 1 initials: |  |
| Rater 2 initials: |  |
| Additional comments, if Poor – why? |  |
