# Supplementary material for: Prognostic Impact and Prevalence of Cachexia in Patients With Heart Failure: A Systematic Review and Meta‐Analysis
Source: J Cachexia Sarcopenia Muscle. 2024 Oct 30;15(6):2536–43. doi: 10.1002/jcsm.13596 (PMC11634528; doi:10.1002/jcsm.13596)
Supplement: Supplementary file 11 — Table S7 Summary of findings table for the impact of cachexia on all‐cause mortality in patients with HF. [file JCSM-15-2536-s003.docx]

| **Table S7.** Summary of findings table for the impact of cachexia on all-cause mortality in patients with HF. | | | | |
| --- | --- | --- | --- | --- |
| **Impact of cachexia on all-cause mortality in patients with heart failure** | | | | |
| **Patient or population: P**atients with heart failure.  **Exposure:** Cachexia  **Comparison: N**o cachexia | | | | |
| Outcomes | Relative effect (95% CI) | № of participants (studies) | Certainty of the evidence (GRADE) | Comments |
|  |  |  |  |  |
| **All-cause mortality** | **HR 1.60** (1.31 to 1.95) | 2252 (4 cohort studies) | ⨁⨁⨁◯ Moderate^a,b^ | Cachexia defined by Evans' criteria likely increases all-cause mortality among patients with heart failure. |
| **CI:** confidence interval; **HR:** hazard ratio | | | | |
| **GRADE Working Group grades of evidence** **High certainty:** we are very confident that the true effect lies close to that of the estimate of the effect. **Moderate certainty:** we are moderately confident in the effect estimate: the true effect is likely to be close to the estimate of the effect, but there is a possibility that it is substantially different. **Low certainty:** our confidence in the effect estimate is limited: the true effect may be substantially different from the estimate of the effect. **Very low certainty:** we have very little confidence in the effect estimate: the true effect is likely to be substantially different from the estimate of effect. | | | | |

#### Explanations

a. Downgraded for risk of bias: only one study was categorized as "good" quality, two as "moderate," and one as having a high risk of bias due to significant methodological concerns. Serious source of bias included mainly lack of precise description of the assessment of components of cachexia definition (such as fatigue, anorexia, decreased muscle strength) and unclear method of death ascertainment.

b. Publication bias could not be assessed due to the limited number of studies included in the review, restricting the ability to conduct a meaningful analysis.
