# Supplementary material for: Prognostic Impact and Prevalence of Cachexia in Patients With Heart Failure: A Systematic Review and Meta‐Analysis
Source: J Cachexia Sarcopenia Muscle. 2024 Oct 30;15(6):2536–43. doi: 10.1002/jcsm.13596 (PMC11634528; doi:10.1002/jcsm.13596)
Supplement: Supplementary file 12 — Table S8 Summary of findings table for the risk of cachexia among patients with HF. [file JCSM-15-2536-s004.docx]

| **Table S8.** Summary of findings table for the risk of cachexia among patients with HF. | | | | |
| --- | --- | --- | --- | --- |
| Risk of cachexia among patients with heart failure | | | | |
| **Patient or population:** Patients with heart failure. | | | | |
| Disease | **Prevalence** (95% CI) | № of participants (studies) | Certainty of the evidence (GRADE) | Comments |
| Cachexia assessed with: Evans' criteria | 31% (21%-43%) | 2862 (10 observational studies) | ⨁⨁◯◯ Low^a,b^ | Cachexia may occur frequently among patients with heart failure. |
| **CI:** confidence interval | | | | |
| **GRADE Working Group grades of evidence** **High certainty:** we are very confident that the true effect lies close to that of the estimate of the effect. **Moderate certainty:** we are moderately confident in the effect estimate: the true effect is likely to be close to the estimate of the effect, but there is a possibility that it is substantially different. **Low certainty:** our confidence in the effect estimate is limited: the true effect may be substantially different from the estimate of the effect. **Very low certainty:** we have very little confidence in the effect estimate: the true effect is likely to be substantially different from the estimate of effect. | | | | |

#### Explanations

a. Downgraded for high risk of bias: four out of ten studies were regarded as having high risk of bias

b. Downgraded for inconsistency: confidence intervals among studies largely do not overlap
